# Influence of PM_1_ and PM_2.5_ on lung function parameters in healthy schoolchildren—a panel study

**DOI:** 10.1007/s11356-016-7605-1

**Published:** 2016-09-15

**Authors:** A. Zwozdziak, I. Sówka, E. Willak-Janc, J. Zwozdziak, K. Kwiecińska, W. Balińska-Miśkiewicz

**Affiliations:** 1 Faculty of Environmental Engineering, Wroclaw University of Technology, Wroclaw, Poland; 2 Department of Paediatrics, Allergology and Cardiology, Wroclaw Medical University, 50-368 Wroclaw, ul. Chałubińskiego 2a, Wroclaw, Poland; 3 Institute of Meteorology and Water Management, National Research Institute, Warsaw, Poland

**Keywords:** Indoor PM pollution, School, Children, Panel study, Lung function

## Abstract

To evaluate lung function responses to short-term indoor PM_1_ and PM_2.5_ concentrations, we conducted a panel study of healthy schoolchildren aged 13–14 years. The following lung function parameters FVC, FEV_1_, PEF, and mid expiratory flows MEF_25_, MEF_50_, and MEF_75_ were measured in 141 schoolchildren of the secondary school in Wroclaw, Poland in years 2009–2010. On days when spirometry tests were conducted, simultaneously, PM_1_ and PM_2.5_ samples were collected inside the school premises. Information about differentiating factors for children including smoking parents, sex, living close to busy streets, dust, mold, and pollen allergies were collected by means of questionnaires. To account for repeated measurements, the method of generalized estimating equations (GEE) was used. The GEE models for the entire group of children revealed the adverse effects (*p* < 0.05) of PM_1_ and PM_2.5_. Small differences in effects estimates per interquartile range (IQR) of PM_1_ and PM_2.5_ on MEF_25_ (5.1 and 4.8 %), MEF_50_ (3.7 and 3.9 %), MEF_75_ (3.5 and 3.6 %) and FEV_1_ (1.3 and 1.0 %) imply that PM_1_ was likely the component of PM_2.5_ that might have a principal health effect on these lung function parameters. However, the reduction of FVC and PEF per IQR for PM_2.5_ (2.1 and 5.2 %, respectively) was higher than for PM_1_ (1.0 and 4.4 %, respectively). Adjustment for potential confounders did not change the unadjusted analysis.

## Introduction

Atmospheric particulate matter (PM) is the main component of air pollution in urban areas and has a significant impact on human health. The principal health effects include premature mortality, respiratory and cardiovascular diseases, and changes in lung function (WHO, 2005, WHO WHO, 2005a, WHO 2007). Children are more vulnerable to air pollution, because of greater ventilation rate per body weight and pulmonary surface area as compared to adults (Ginsberg et al. 2005; Bateson and Schwartz 2008). Deep breathing pulls air pollutants faster and further into the lungs—bypassing initial areas of deposition. The study by Ginsberg et al. (2005) has reported that the pulmonary region of the lung has slower clearance, thus particles remain there longer, meaning that the particle dose can be two- to fourfold higher among young children as compared to adults (Ginsberg et al. 2005).

The effect of air pollutants on lung function depends on the type of pollutant and its ambient concentration, duration of exposure and the total lung ventilation period of exposed individuals (D’Amato et al. 2010). Inhalable particles that can reach the lower airways are classified into three size fractions: PM_10_ (diameter ≤ 10 μm), PM_2.5_ (diameter ≤ 2.5 μm) and PM_1_ (diameter ≤ 1 μm). Particles included in PM_10_ but larger than 8 μm mostly remain in pharynx, larynx and trachea. Approximately 20 % of 10-μm particles penetrate through the extrathoracic airways and into the lower respiratory tract (Brown et al. 2013). Particles of 5–10 μm size reach upper parts of the bronchi, where they are removed in the process of mucociliary clearance, provided that the mucosa and cilia are intact (D’Amato et al. 2010). Unremoved particles affect the bronchial epithelium and muscularis mucosae of all parts of the bronchi, hence their importance should be considered with particular care. Although, the standard classification into PM_10_, PM_2.5_, and PM_1_ is broadly accepted, the complexity of the problem with classification of particles is still under discussion since the deposition of particle in lungs depends on various factors including sex, age, breathing habits, and activities (Brown et al. 2013).

Recent study suggests that 50 % of particles less than 4 μm in diameter penetrate into the lower respiratory tract in children (Brown et al. 2013). Other studies proved that particles with diameters equal or smaller than 2.5 μm (PM_2.5_) reach the alveoli and up to 50 % of them may remain in the lung tissue (Valavanidis et al. 2008). Fine PM can penetrate deep into the airways and induce alveolar inflammation, which is responsible for release of mediators favoring acute episodes of respiratory diseases (Schwartz 1992). Due to deep deposition they are removed very slowly, increasing the chances of causing cell damage (See and Balasubramanian 2008). Ultrafine particles are usually removed during the exhalation.

The association between exposure to ambient PM and reduced lung function parameters has been reported in many studies (Jedrychowski et al. 1999; Horak et al. 2002; Roy et al. 2012, Badyda et al. 2015). In recent decades, numerous panel studies of the influence of PM pollution on children’s lung function and respiratory symptoms were conducted and showed greater adverse effects of PM_2.5_ than PM_10_ on respiratory health outcomes in children (Ward and Ayres 2004; Li et al. 2012). The health effects of PM_1_ exposure were examined in only a few studies (Yu et al. 2000; Mar et al. 2004, Moshammer et al. 2006). Yu et al. (2000) found that the same-day PM_1_ concentrations were associated with asthma symptoms in children, Mar et al. (2004) identified a strong association between cough and both PM_1_ and PM_2.5_. In the studies of Moshammer et al. (2006), the changes per IQR were in the magnitude of 1 % for PM_1_.

The aim of this study was to assess the short-time effects of indoor dust particles with different aerodynamic diameters (PM_1_, PM_2.5_) on lung function data in healthy school children. While two indicators were considered, the analysis addressed whether a particular PM indicator better fitted to the data. Thus, the important issue was to identify the PM component with an acute effect.

## Material and methods

### Study population

The study was performed in the secondary school located in Wroclaw, a city in south-western Poland with population of approximately 700,000, near busy intersections (ca. 150 m). The school was chosen as the measurement site because it was located in a heavily populated residential area and the majority of the students resided in the area. Additionally, the school had sufficiently large student population. We reasoned that the children attending the school located in city center were likely to be exposed to indoor pollutants that infiltrate from outdoors and potentially may have negative impact on their health.

Similarly to other buildings in the area, the school was erected based on construction standards from the beginning of the last century, including no provisions for air conditioning system. Inside the building there is a huge patio (30 m long, 15 m wide, and 10 m high) covered with a glass roof. Corridors and classroom’s doors are placed along the perimeter of the patio on each floor. The classrooms are naturally ventilated via the doors, which are opened to the corridors during breaks. Thus, the air contained within the patio may be considered to be representative of the classroom condition.

The parents of the group of examined children (13–14 years old) had to provide their written informed consent for the participation of their child in the study and to fill out a questionnaire containing questions on respiratory and allergic diseases and home environment. Children with diagnosed bronchial asthma or other chronic respiratory diseases were excluded from the study. At the beginning, the study included 179 schoolchildren between 13 and 14 years old. We planned to test each healthy child ten times during the school year (2009/2010); however, as a result of school absences due to various reasons or symptoms of a common cold present on testing days, not all children were examined the intended number of times. In the end, our data base included 141 children with complete pulmonary function data from seven days during the school year 2009–2010.

Bioethics Committee of Wroclaw Medical University approval was obtained to conduct research in schoolchildren.

### Lung function measurements

The children underwent lung function tests with the use of Blue Spiro portable spirometer (Micro 500; Medisoft Group). FVC, FEV_1_, PEF, and mid expiratory flows (MEF_25_, MEF_50_, MEF_75_) were obtained following the protocol of the American Thoracic Society (ATS) and the European Respiratory Society (ERS) (Miller et al. 2005 *)*. All lung function data were expressed as percentage of the predicted normal values according to the ECCS/ERS reference equation.

Specifically, FVC, FEV_1_, PEF, and mid expiratory flows (MEF_25%_, MEF_50%_, and MEF_75%_) were measured in sitting position after at least 15 min of rest, while wearing a nose clip. A spirometry test was performed by trained professional medical staff and held in accordance with ATS/ERS procedures, except for the minimum exhalation time of 6 s, which is not feasible for most of the children (Arets et al. 2001). The results of spirometry were acceptable when FET was at least 3 s.

For each child, the aim was to get at least three acceptable maneuvers; however a maximum of eight attempts were allowed. Body weight and height were measured during the medical examination using calibrated measuring equipment.

The tests were performed for all participating children at the same time of day, between 9:00 and 14.00, once a month from December 2009 to October, 2010 (no measurements in July and August due to school holidays).

### Short-term air pollution exposure assessment

The spirometry measurements were carried out in the hall of the building, which forms a part of the patio. The 8 h average of PM_1_ and PM_2.5_ (08:00–16:00) was considered in evaluation of lung function responses. These values included the lower concentrations during the lessons and the highest concentrations during the breaks. We assumed that short-term respiratory effects could be observed at the time of elevated levels of PM. Thus, the teaching hours average should represent the period of higher concentrations compared to the other times of the day. However, to characterize the overall indoor air pollution, PM_1_ and PM_2.5_ concentration measurements were performed on daily basis as 8 h means (08:00–16:00) and 16 h means (16:00–08:00) during the weekdays (from Monday to Friday) when lung functions were tested on the same days. Two Harvard cascade impactors (MS&T Area Samplers, AirDiagnostics and Engineering, Inc., Harrison, ME, USA) were used simultaneously. The pumps (Air Diagnostics and Engineering, model SP-280E) were set at airflow of 23 dm^3^/min for PM_1_ and 10 dm^3^/min for PM_2.5._ The volume of pumped air was controlled by Actaris-type flow meter. The particles were collected by 37 mm diameter Teflon membrane filters (PALLFLEX, TK15-G3 M). All filters were pre- and post-conditioned in a clean room with environmentally controlled temperature and humidity prior to weighing. Weighing was carried out with an electronic microbalance (Santorius M5P 000 V001) with ±1 μg sensitivity and in the 500 mg range. The analysis was carried out in accordance with European PM Marking Standard (NBN-EN-12,341). Before weighing, filters were conditioned for 48 h at 20 °C.

### Covariates

Data on inter-child differences such as smoking parents at the child’s home (SMOKERS), sex (SEX), living on the main city street (STREET), dust allergy (DUST), pollen allergy (POLLEN), molds allergy (MOLD), dampness in the child’s home (DAMP), and traffic nuisance due to the passage of trucks (TRAFFIC) were collected by means of questionnaires. The environmental conditions such as temperature or humidity were nearly constant inside the school during the study period; therefore, these variables were not considered as potential covariates. Air temperature, humidity, and air velocity were checked using Mini HydroThermo-Anemometr (Extech Instruments) four times a day throughout the sampling periods. The indoor temperature varied slightly from 18 to 21 °C, regardless of the season; relative humidity ranged between 31 and 54 %; air velocity was below the detection limit (0.5 m/s).

### Statistical analysis

We used lung function parameters (outcome variables) as dependent variables to analyze their response to PM pollution. An outcome variable is a percent of the value rather than an absolute value. Thus, lung function values were log-transformed because of expected multiplicative effects (Moshammer et al. 2006). To account for repeated measurements, the method of generalized estimating equations (GEE – single pollutant model) was used with an autoregressive working correlation matrix. The same group of schoolchildren (141) was tested seven times in different exposures to indoor PM. GEE is one of the most frequently used statistical method in such panel studies (Li et al. 2012). Data entry and analyses were done with R package geepack Version 3.1 (http://www.r-project.org/). Statistical significance was determined using a *p*-value of 0.05.

All effect estimates from GEE models (β coefficients, 95 % CI) were transformed into percent change of lung function parameters measured per interquartile range (25–75 %; IQR) of the respective PM pollutant using the formula (e^β*IQR^ – 1)×100.

A univariate GEE model was also run to provide information for inter-child differences and to examine the impact of each covariate on each lung function parameter. Covariates were defined as dichotomous variables.

Finally, we performed a multivariate approach of GEE for each PM pollutant and those covariates that were significantly (*p* < 0.05) associated with at least one of the outcome variables in univariate models.

## Results

### Characteristic of the study population

**Fig. 1:**
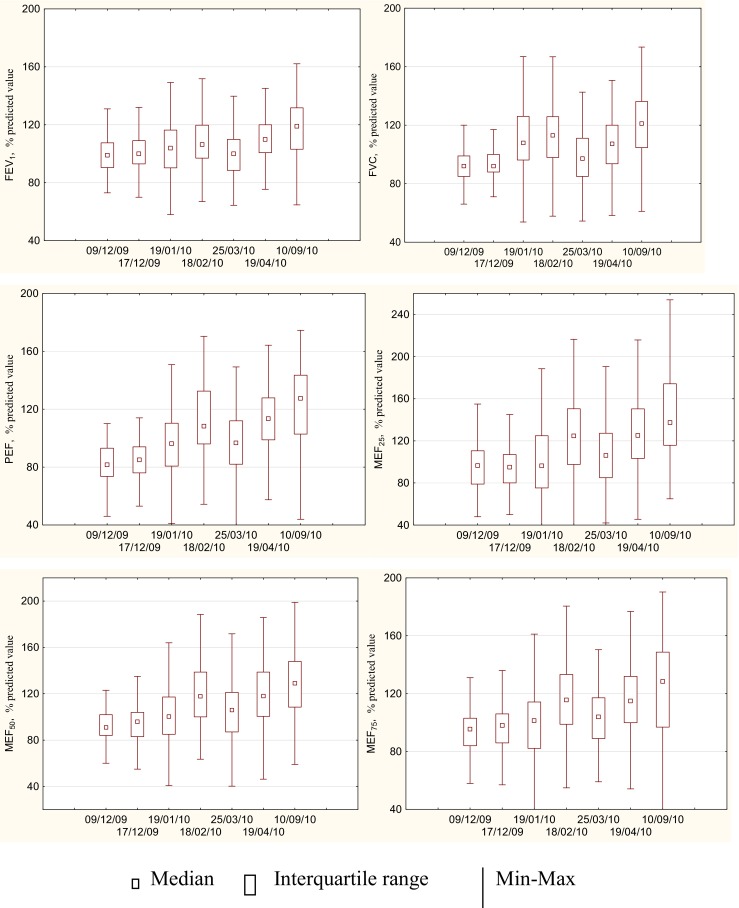
Descriptive statistics of lung function parameters among schoolchildren (*n* = 141) in following testing days

Characteristics of the study population and the distribution of lung function parameters are presented in Table 1 and in Fig. 1.

**Table 1:** Population characteristics (*n* = 141)

Variable	*N*	Percent (%)
Female SEX	91	64.5
Smoking at child’s home SMOKERS	51	36.2
Dampness at child’s home DAMPNESS	5	3.5
Living on the main city street STREET	57	40.4
Pollen allergy POLLEN	35	24.8
Dust allergy DUST	24	17.0
Mold allergy MOLD	3	2.1
Passage of trucks nuisance TRAFFIC	54	38.2

Using the dependent *t* test (the same group of children was tested repeatedly over time), we compared the means of lung function parameters collected on different testing days to detect whether there were any statistically significant differences between these means. Before doing this, we made sure that our data met assumptions of the test. With *p*-values greater than 0.05 (*p* > 0.05), we could conclude that there were no significant differences between the means of PEF and MEF in December and January, and March, as well as in February and April, and September which essentially states that there were significant differences in means between December and February, and April, and September. There were significant differences in means for FEV_1_ between December and only April, and September; for FVC between December and all remaining testing days.

Using the univariate GEE approach, we estimated associations for FEV1, FVC, PEF, MEF_25_, MEF_50_, MEF_75_ and potentially cofounders: SEX, SMOKING, DAMPNESS, STREET, DUST, POLLEN, MOLD, and TRAFFIC (Table 2). Associations between all six lung function parameters and SMOKING, DAMPNESS, POLLEN, and MOLD were not statistically significant. Negative and significant associations were observed for SEX (boys). Associations showed the discrepancy for STREET, DUST, and TRAFFIC. There were significant (*p* < 0.05) negative associations between PEV_1_ and PEF and children feeling traffic nuisance, TRAFFIC, and between FEV and MEF_50_ and children living near heavy traffic roads, STREET, and between MEF_50_ and children allergic to house dust, DUST. Nevertheless, all these covariates, e.g., SEX, STREET, DUST, and TRAFFIC were considered in a multivariate approach of GEE for each PM pollutant.

**Table 2:** Effects estimates (*β*, 95 % CI) and probability level (*p*) for the associations of logFEV1, logFVC, logPEF, logMEF_25_, logMEF_50_, logMEF_75_ and potentially confounders (italics with *p* < 0.05)

	FEV_1_		FVC		PEF		MEF_25_		MEF_50_		MEF_75_	
	*β* (95%CI)	*P*	*β* (95%CI)	*p*	*β* (95%CI)	*P*	*β* (95%CI)	*P*	*β* (95%CI)	*p*	*β* (95%CI)	*p*
SEX (boys)	*-0.027* *(−0,045;* *-0.009)*	*<0.05*	*-0.039* *(−0.060,* *- 0.019*	*<0.05*	*-0.028* *(−0.048,* *-0.007)*	*<0.05*	*-0.058* *(−0.087,* *-0.029)*	*<0.05*	*-0.046* *(−0.070,* *-0.022)*	*<0.05*	*-0.057* *(−0.078,* *-0.035)*	*<0.05*
SMOKING	-0.012 (−0.030, 0.007)	0.22	-0.010 (−0.031, 0.011)	0.35	-0.007 (−0.027, 0.015)	0.54	-0.011 (−0.041, 0.015)	0.48	-0.012 (−0.037, 0.013)	0.95	-0.007 (−0.030, 0.016)	0.56
DAMPNESS	0.013 (−0.027, 0.053)	0.53	0.015 (−0.032, 0.061)	0.53	0.030 (0.000, 0.060)	0.06	0.013 (−0.046, 0.073)	0.67	0.020 (−0.027, 0.067)	0.41	0.012 (−0.027, 0.052)	0.55
STREET	-0.014 (−0.031, 0.003)	0.10	-0.014 (−.0033, 0.006)	0.16	*-0.02* *(−0.040,* *-0.001)*	*0.04*	-0.014 (−0.042, 0.015)	0.34	*-0.030* *-0.053,* *-0.006*	*0.02*	-0.022 (−0.045. 0.000)	0.05
DUST	0.010 (−0.012, 0.033)	0.37	0.008 (−0.018, 0.035)	0.52	0.016 (−0.009, 0.041)	0.20	0.012 (−0.026. 0.049)	0.55	*0.037* *(−0.009, 0.065)*	*0.01*	0.027 (−0.001, 0.056)	0.06
POLLEN	-0.002 (−0.021, 0.016)	0.80	-0.006 (−0.028, 0.015)	0.56	0.003 (−0.019, 0.025)	0.78	0.011 (−0.020, 0.043)	0.49	0.004 (−0.020, 0.028)	0.73	0.005 (−0.021, 0.031)	0.72
MOLD	-0.002 (−0.030, 0.026)	0.89	-0.002 (−0.050, 0.046)	0.93	-0.017 (−0.062, 0.041)	0.68	-0.029 (−0.083, 0.025)	0.30	-0.003 (−0.043, 0.038)	0.89	0.000 (−0.058, 0.057)	0.99
TRAFFIC	*-0.028* *(−0.055,* *-0.001)*	*0.04*	-0.014 (−0.045, 0.018)	0.39	*-0.034* *(−0.066,* *-0.002)*	*0.04*	0.000 (−0.033, 0.034)	0.98	0.000 (−0.034, 0.033)	0.98	-0.030 (−0.065, 0.006)	0.11

### 2.2. Indoor PM_1_, and PM_2.5_ concentrations

**Fig. 2:**
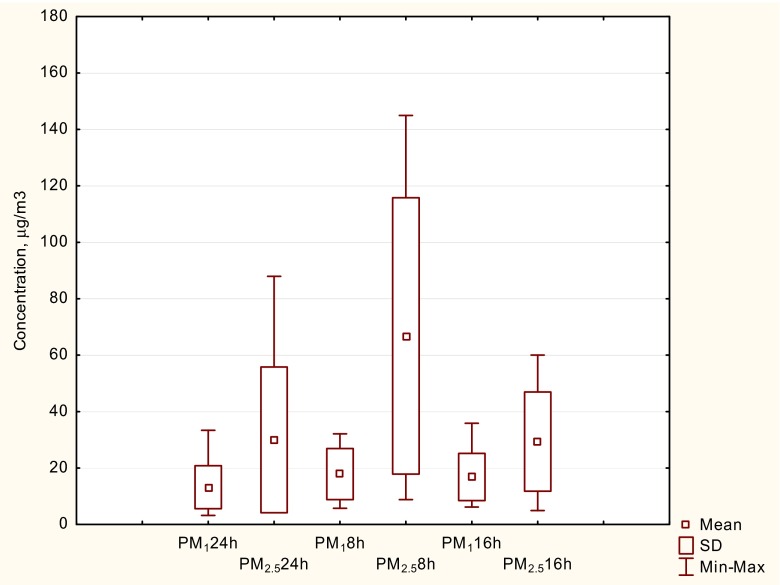
A box-and-whisker plot of 24 h (PM2 4 h ), 8 h (PM8 h) and 16 h (PM1 6 h) data set (the whole sampling period, covering these weeks, when the children were tested in a single day)

The means, standard deviations and IQRs of the concentrations for the spirometry days (test days) are shown in Table 3. How did these values relate to the average concentrations in school? Thus, 8-h mean levels at the testing days (Table 3) were comparable to daily mean PM for the whole sampling period, covering the weeks, when the children were tested in a single day (Fig. 2). It follows that the short-term effects of PM on lung function were investigated across a typical range of school environmental conditions (and hence different exposure of PM_1_ and PM_2.5_).

**Table 3:** Means, standard deviations and interquartile ranges (IQR) of short-term (8-h average) exposures to PM inside the school building during the spirometry days (*n* = 7)

PM fraction	Mean(standard deviation), μg/m^3^	Interquartile range (IQR), μg/m^3^
PM1	22 (8)	9
PM2.5	67 (43)	41

Figure 2 shows the box plots representing the distribution of 24 h (PM2 4 h ), 8-h (PM8 h)n and 16-h (PM1 6 h) mean concentrations of PM_1_ and PM_2.5_ inside the school hall during the whole sampling period. Nightly concentrations were the indicators for the strength of indoor sources during the teaching hours. Thus, the findings indicate that indoor dust sources caused of elevated PM_2.5_ concentrations during daytime as compared to nighttime (ca. three times higher). PM_1_ concentrations reached comparable levels for both sampling periods. Additionally, there were significant but not strong correlation between PM_1_ and PM_2.5_ indoor concentrations (*R* ^2^ = 0,34; *p* = 0.045), implying that it will be possible to identify the PM component that may have the adverse effect. The small *R* ^2^ value means that there were likely different sources of origin for PM_2.5_ and PM_1_. Details of the contributions of outdoor and indoor sources to the indoor concentrations of PM_1_ and PM_2.5_ have been described by Zwoździak et al. 2013, 2014.

### 2.3. Lung function responses to PM pollution

The associations between indoor 8 h averages of PM_1_ and PM_2.5_ concentrations and lung function parameters, FEV_1_, FVC, PEF, MEF_25_, MEF_50_, and MEF_75_, within the schoolchildren population estimated by the single pollutant GEE model is shown in Table 4. In all analyses, PM_1_ and PM_2.5_ were significant predictors (*p* < 0.05); the negative associations suggested decreases in lung function with increasing exposure. In general, the comparison of effect estimates suggests a greater effect for PM_1_ compared to PM_2.5_. Overall effect estimates were small only for FEV_1_, in the magnitude of 1 % per IQR. The strongest changes were seen for PEF and in maximal expiratory flow in small airways (MEF’s), in the magnitude of 4–5 % per IQR. As PM_1_ was a part of PM_2.5_ (from 50 to 82 %, on average 70 % of PM_2.5_ inside school during testing days) and very small differences in effects estimates for PM_1_ and PM_2.5_ observed (except for FVC and PEF) implied that mainly PM_1_ may have the adverse effect, while exposure to dust PM_2.5_ did not substantially reduce lung function as compared to PM_1_, particularly in maximal expiratory flow in small airways (MEF).

**Table 4:** Percent change (%) and 95 % CI in lung function parameters per interquartile change range (IQR) of the respective PM pollutant (results from single pollutant GEE models, *p* < 0.05)

PM fraction	FEV1		FVC		PEF		MEF25%		MEF50%		MEF75%	
	%/ IQR	95%CI	%/ IQR	95%CI	%/ IQR	95%CI	%/ IQR	95%CI	%/ IQR	95%CI	%/ IQR	95%CI
PM_1_	−1.3	−1.8, −0.8	−1.0	−1.5, −0.5	−4.4	−5.0, −3.7	−5.1	−5.8, −4.5	−3.7	−4.5, −3.0	−3.5	−4.2, −2.8
PM_2.5_	−1.0	−1.4, −0.6	−2.1	−2.6, −1.6	−5.2	−5.7, −4.7	−4.8	−5.4, −4.2	−3.9	−4.5, −3.2	−3.6	−4.2, −3.0

Final results from the multivariate GEE model are presented in Appendix (Table 5) because the estimated percent change of a given outcome variable per IQR increase in the exposure to each air pollutant, controlling for sex (SEX), living close to busy streets (STREET), dust allergy (DUST) and traffic nuisance (TRAFFIC) remained negligible changed compared to the single pollutant analysis. It indicates that adjustment for potential confounders did not change the unadjusted analysis and small changes of lung function parameters after exposure to PM pollution were observed, despite personal home environment and allergies. We adjusted statistical parameters for known potential confounders (Table 2), but the possibility of confounding by other factors still exists (including temperature, season, and other air pollutants).

## Discussion

The main interest of the investigation was whether short-term PM (PM_1_, PM_2.5_) concentrations had an impact on lung function parameters of healthy schoolchildren. The measured daily 8-h mean PM_2.5_ concentrations inside the school building were nearly two times higher than the calculated 24-h means. Air quality standards are based on outdoor PM_2.5_ concentrations. There is no legal limit value for outdoor PM_1_ and indoor PM_2.5_ and PM_1_ yet. The mean 24-h indoor concentration of PM_2.5_ was 37 μg/m^3^ in the school under investigation. This is consistent with several other studies that have shown that PM_2.5_ concentrations in classrooms were high and exceeded the World Health Organization Air Quality Guidelines (WHO 2005a) for the ambient air (Ekmekcioglu and Keskin 2007; Diapouli et al. 2008; Oeder et al. 2012). The short-term WHO AQG for PM_2.5_ is 25 μg/m^3^ (24-h means). Notably, children usually spend approximately 6–8 h in school and are therefore exposed to high levels of PM.

The results presented in Table 4 suggest that high indoor PM levels can lead to reduced values of lung function parameters: FEV_1_, FVC, PEF, MEF_25_, MEF_50_, and MEF_75_. The associations were statistically significant (*p* < 0.05) for PM fractions studied, however differences in effect estimates were observed both for the PM size and measured outcome variables. The changes per IQR were in the range from 1 to 2 % only for FEV_1_ and FVC. The strongest changes per IQR, from 3.5 to 5.2 %, were observed for PEF and MEF_25_, MEF_50_ and MEF_75_. As compared to the study carried out in Linz, Austria (Moshammer et al. 2006), the changes in spirometric parameters of schoolchildren were higher in our study. In Austria, 163 healthy children aged 7–10 were tested once a month throughout 1 year. The spirometric parameters were tested in relation to 8 h mean concentration of PM_1_ recorded at a nearby monitoring station. Using the GEE method, the lung function deficits per IQR were in the magnitude of 1 % for PM_1_. The interquartile range was 10.9 μg/m^3^, in our study 9.0 μg/m^3^, respectively but the 8 h mean was 12.3 μg/m^3^, i.e., lower than in our study – 22.0 μg/m^3^.

The study of Chan et al. (2015) has provided evidence that exposure to current levels of ambient PM_2.5_ in Taiwan was associated with reductions in children’s lung function (FEV_1_, FVC). They performed spirometry in 1494 healthy children aged 6–15 years from 44 schools. Air pollution data were obtained from air monitoring stations within 1 km of the schools. Authors concluded that sub-chronic exposure to ambient PM_2.5_ (mean concentration: 38.6 μg/m^3^) reduces lung capacity in children aged 6–15 years and may induce additional airway obstructive patterns of lung function in children aged 6–10 years. In contrast, Epton et al. (2008) who studied the effect of ambient particulate air pollution on the respiratory function of male schoolchildren (93 students) in New Zealand concluded that only asthmatic children showed small effects of high PM_10_ levels on FEV_1_ and PEF. No significant effects were observed for healthy children. The majority of PM_10_ pollution was in the PM_2.5_ range and indoor pollution levels were similar to outdoor.

A review paper of panel studies in children did not provide a clear association of lung function with increasing PM pollution (Ward and Ayres 2004). Ward and Ayres 2004 concluded that a considerable diversity of results was observed. However, a small adverse effect of PM_2.5_ for PEF was shown. Healthy children appeared to be more affected by PM levels than those with diagnosed respiratory symptoms. This finding was in contrast to a literature review by Li et al. (2012). They concluded that more serious adverse effects of PM on lung function parameters were observed for asthmatic children.

In many panel studies, PEF and FEV_1_ were the most frequently measured parameters to assess the effects of PM_2.5_ or PM_10_ pollution on children’s lung function (Ward and Ayres 2004; Li et al. 2012; Jacobson et al. 2012; Chan et al. 2015). Despite the fact that the smallest fraction penetrates the deepest into the airways, the health effects of PM_1_ on lung function in children have been poorly investigated. Thus, the strength of our study was that two fractions of PM (PM_1_ and PM_2.5_) and six different spirometry parameters (FEV_1_, FVC, PEF, MEF_25_, MEF_50_, and MEF_75_) were measured simultaneously and their average associations within the schoolchildren population were estimated. We observed negative impact of both indoor PM_1_ and PM_2.5_ on lung function parameters of healthy schoolchildren. It should be emphasized that indoor PM_1_ accounted on average for 70 % of PM_2.5_ mass concentrations during testing days.

Overall, our finding of a decrease in lung function with increasing exposure to PM_1_ and PM_2.5_ is consistent not only with other short term studies, but also with long term studies such as cohort studies in Europe, ESCAPE study (Gehring et al. 2013), which have confirmed the adverse effect of PM_2.5_ pollution. For example, statistically significant decreases in PEF, 0.8 % per 5 μg/m^3^ increase in PM_2.5_ was found in a Norwegian population study (Oftedal et al. 2008).

Important limitations to our study must be noted. Firstly, the sample size was fairly small: individual level observations of lung function in 141 schoolchildren and PM pollution records were collected for 7 days, although, across varied exposure levels of PM_1_ and PM_2.5_. Thus, attempts at generalization should be done with caution. Secondly, the observed effects may be biased by the assumption of the exposure time and teaching hours. The 8-h mean PM concentration on the days of spirometry measurements, was assumed to be suitable for this kind of study. To assess a group level function: concentration- response (impact on children respiratory system as a whole, we did not consider this relationship for individual child), data are needed that characterize concentrations when children are at school. In short-term effect studies, the largest effects have been often reported for air pollution levels on the day when the lung function measurements were performed or on the days preceding the examination (Gehring et al. 2013). We realize that the best estimates require the use of monitoring devices that can be carried by pupils. This is the direction for our further studies. Further research is needed to examine the lag effects of PM pollution on children’s lung function and interactive effects between PM concentrations and indoor humidity.

## Conclusions

Exposure to elevated PM concentrations causes a decrease in the lung function parameters in healthy schoolchildren resulting in poorer spirometry results. According to WHO (http://www.euro.who.int/) there is no evidence of a safe level of exposure or a threshold below which no adverse health effects of PM occur.

We observed the greater effect for PM_1_ compared to PM_2.5_ on the lung function parameters. PM_1_ fraction is likely to be the better indicator for acute effects than PM_2.5_ fraction indoor (at the school) and involves pronounced changes in schoolchildren lung function mainly in small airways. PM_1_ mass concentrations are not routinely monitored at most air pollution monitoring stations but may be more important for health effects than bigger size PM fractions.

The negative impact of indoor PM_1_ and PM_2.5_ pollution on healthy children lung function requires an effective indoor air quality management program, which is necessary to reduce children’s health risks to a minimum.

## Appendix

**Table 5:** Adjusted for SEX, DUST, STREET, and TRAFFIC associations between 8-h average PM levels and lung function parameters (results from multivariate GEE models, *p* < 0.05)

PM fraction	FEV1		FVC		PEF		MEF25%		MEF50%		MEF75%	
	%/ IQR	95%CI	%/ IQR	95%CI	%/ IQR	95%CI	%/ IQR	95%CI	%/ IQR	95%CI	%/ IQR	95%CI
PM1	−1.3	−1.7, −0.8	−1.0	−1.6, −0.5	−4.4	−5.0, −3.7	−5.1	−5.9, −4.5	−3.8	−4.5, −3.0	−3.6	−4.3, −2.9
PM2.5	−1.0	−1.4, −0.6	−2.1	−2.6, −1.6	−5.2	−5.7, −4.7	−4.7	−5.3, −4.1	−3.9	−4.5, −3.2	−3.5	−4.1, −2.9

Abbreviations:

ECCS - European Community of Coal and Steel
